# Protein Kinase RNA-Like Endoplasmic Reticulum Kinase-Mediated Bcl-2 Protein Phosphorylation Contributes to Evodiamine-Induced Apoptosis of Human Renal Cell Carcinoma Cells

**DOI:** 10.1371/journal.pone.0160484

**Published:** 2016-08-02

**Authors:** Wen-Shin Wu, Chih-Chiang Chien, Yen-Chou Chen, Wen-Ta Chiu

**Affiliations:** 1 Graduate Institute of Medical Sciences, Taipei Medical University, Taipei, 110, Taiwan; 2 Department of Biochemistry and Molecular Cell Biology, School of Medicine, College of Medicine, Taipei Medical University, Taipei, 110, Taiwan; 3 Department of Nephrology, Chi-Mei Medical Center, Tainan, Taiwan; 4 Department of Food Nutrition, Chung Hwa University of Medical Technology, Tainan, Taiwan; 5 Cancer Research Center and Orthopedics Research Center, Taipei Medical University Hospital, Taipei, 110, Taiwan; 6 Department of Neurosurgery, Taipei Municipal Wan-Fang Hospital and Graduate Institute of Clinical Medicine, Taipei Medical University, Taipei, 110, Taiwan; Tel-Aviv University, ISRAEL

## Abstract

We investigated the anticancer mechanism of evodiamine (EVO) against the viability of human A498 renal cell carcinoma (RCC) cells in vitro and in vivo. The in vitro study showed that EVO decreased the viability of A498 cells with the occurrence of apoptotic characteristics such as hypodiploid cells, DNA ladders, chromatin-condensed cells, and cleaved caspase (Casp)-3/poly(ADP ribose) polymerase (PARP) proteins. Pharmacological studies using chemical inhibitors of mitogen-activated protein kinase (MAPK) and phosphoinositide 3-kinase (PI3K) indicated that phosphorylation of the c-Jun N-terminal kinase (JNK) protein participated in EVO-induced cell death of A498 cells, and application of the JNK inhibitor, SP600125 (SP), inhibited EVO-induced cleavage of the Casp-3/PARP proteins and chromatin condensation according to Giemsa staining. EVO disruption of the mitochondrial membrane potential (MMP) with increased protein levels of the phosphorylated Bcl-2 protein (p-Bcl-2) was prevented by JNK inhibitors in A498 cells. A structure-activity relationship study showed that a methyl group at position 14 in EVO was important for its apoptotic effects and increased p-Bcl-2 protein in A498 cells. Furthermore, significant increases in the phosphorylated endoplasmic reticular stress protein, protein kinase RNA-like endoplasmic reticulum kinase (p-PERK at Thr980), by EVO were detected in A498 cells, and the PERK inhibitor, GSK2606414, significantly suppressed EVO-induced apoptosis, p-JNK, p-PERK, and cleaved PARP proteins. The in vivo study showed that EVO significantly reduced RCC growth elicited by a subcutaneous injection of A498 cells, and an increased protein level of p-PERK was observed according to an immunohistochemical analysis. Apoptosis by EVO was also demonstrated in other RCC cells such as 786-O, ACHN, and Caki-1 cells. This is the first study to demonstrate the anti-RCC effect of EVO via apoptosis in vitro and in vivo, and activation of JNK and PERK to induce Bcl-2 protein phosphorylation, which led to disruption of the MMP.

## Introduction

Renal cell carcinoma (RCC) accounts for around 90%~95% of all kidney neoplasms [1, 2] and surgery remains the only definitive treatment for RCC [3]. RCC is highly refractory to conventional therapeutic strategies, including radiotherapy [4], chemotherapy [5], and hormonal therapy [6]. There are five major subtypes of RCC, and clear-cell RCC is very aggressive and the most common histologic subtype [2, 7, 8]. Therefore, development of chemicals with effective inhibitory activity against RCC especially clear-cell RCC growth is an urgent need for treating RCC. Natural products are a source of compounds possessing therapeutic benefits in treating human diseases. Evodiamine (EVO) is one of chemicals in *Evodia rutaecarpa*, and has been shown to perform various biological effects including antitumor, antinociceptive, and vasorelaxant properties were reported [9]. EVO expressed inhibitory effects on tumor cell migration in vitro, and induced cell death in several cell types with little effect on normal human peripheral blood mononuclear cells [10]. Although EVO inhibition of tumor growth was reported, the in vitro and in vivo actions of EVO on the growth of RCC are still unclear.

A disturbance of the balance between cell proliferation and apoptosis results in several pathological conditions including cancers, and several studies indicated that reduction of tumor growth by anticancer agents is mediated by the induction of apoptosis [11–15]. Mitochondrial apoptotic pathways have been shown to participate in apoptosis elicited by various chemotherapeutic agents, supporting their importance cancer treatments [15–19]. Disruption of the mitochondrial membrane potential by chemicals initiates caspase (Casp)-9 activation, which in turn activates effector caspases such as Casp-3 leading to caspase-dependent DNA fragmentation, a characteristic of apoptosis [17, 20]. Bcl-2 family proteins regulate the mitochondrial membrane permeability (MMP) in apoptosis, and both proapoptotic (e.g., Bax, and Bak) and antiapoptotic (e.g., Bcl-2, and Bcl-xL) Bcl-2 family proteins have been identified [21–24]. Previous studies showed that decreased levels of antiapoptotic and/or increased levels of proapoptotic Bcl-2 family proteins were observed with apoptosis of cancer cells under chemical stimulation [15, 24]. It indicates that changes in balance of the Bcl-2/Bax complex leads to an anti- or proapoptotic effect, and increased Bax or decreased Bcl-2 protein may induce loss of the MMP that initiates apoptosis progression [22, 25–27]. Additional evidence suggests that phosphorylation of Bcl-2 may affect its ability to form dimers with other proapoptotic proteins leading to apoptosis. c-Jun N-terminal kinase (JNK) is known to regulate apoptosis through phosphorylation of the Bcl-2 protein[12, 28, 29]. The contributions of JNK and Bcl-2 protein phosphorylation to EVO-induced apoptosis of human renal carcinoma cells are still unclear.

Protein kinase RNA (PKR)-like endoplasmic reticular (ER) kinase (PERK) is a serine/threonine ER kinase, and serves as a sensor in the ER to monitor cell homeostasis[28, 30]. It is still unclear whether PERK positively contributes to cancer progression or plays a role as a significant therapeutic target in cancer treatments. Although EVO induction of apoptosis has been observed in several cancer cells including glioblastoma, colon cancer, and lung cancer cells, the effect of EVO on the viability of human renal cancer cells in vitro and in vivo accordingly with the role of JNK/PERK are still unclear. In this study, we found that EVO reduced viability via apoptosis and accordingly activates JNK and PERK leading to increased phosphorylation of the Bcl-2 protein and disruption of the MMP in human RCC cells. In vivo anti-RCC growth by EVO was identified in athymic nude mice.

## Materials and Methods

### Cell culture

A498 RCC cells were obtained from the American Type Culture Collection (Manassas, VA, USA). Cells were maintained in Eagle's minimum essential medium supplemented with antibiotics (100 U/mL penicillin and 100 U/mL streptomycin), 2 mM L-glutamate, 1.0 mM sodium pyruvate, 1% non-essential amino acids, and 10% heat-inactivated fetal bovine serum (FBS; Gibco/BRL, Grand Island, NY, USA) and maintained in a 37°C humidified incubator containing 5% CO_2_.

### Agents

The chemical reagents of EVO, SP600125, BCIP, 3-(4,5,-dimethylthiazol)-2-yl-2,5-diphenyltetrazolium bromide (MTT), and NBT were obtained from Sigma Chemical (St. Louis, MO, USA). Antibodies of α-tubulin, poly (ADP ribose) polymerase (PARP), Casp-3, Casp-9, Bcl-2, and Bax were obtained from Santa Cruz Biotechnology (Santa Cruz, CA, USA). Antibodies of total (t) and phosphorylated (p) MAPK (total extracellular signal-regulated kinase (ERK)/p-ERK and total c-Jun N-terminal kinase (JNK)/p-JNK) proteins were obtained from Cell Signaling Technology (Danvers, MA, USA). Other chemicals not mentioned above were obtained from Sigma Chemical. EVO-related chemicals were based on the coupling of 3,4-dihydro-β-carboline with substituted N-alkyl isatoic anhydride in pyridine. 3,4-Dihydro-β-carboline was prepared by reacting tryptamine with ethyl formate, followed by intramolecular ring closure in the presence of POCl_3_. Their purities were >95% when analyzed by high-performance liquid chromatography (HPLC) [8, 13].

### MTT assay

Cell viability was assessed by MTT staining as described previously [31, 32]. Briefly, cells were plated at a density of 10^5^ cells/well in 24-well plates. At the end of treatmeCell viability was measured using the MTT method as described previously [31, 32]. 10^5^ cells/well in 24-well plates, when the end of treatment, 20 μl of the tetrazolium compound, MTT, was added. After incubation for 4 h at 37°C, the supernatant was removed, and 200 μl of DMSO was placed in each well to dissolve the tetrazolium crystals. Finally, the absorbance at a wavelength of 595 nm was recorded using an enzyme-linked immunosorbent assay (ELISA) plate reader.

### Western blotting

Total cellular extracts (30 μg) were prepared and separated on 8% sodium dodecylsulfate (SDS)-polyacrylamide mini gels for PARP detection and 12% SDS-polyacrylamide minigels for Casp-3, Casp-9, the Bcl-2 family, t-ERK, p-ERK, and α-tubulin detection, and transferred to Immobilon polyvinylidene difluoride membranes (Millipore, Bedford, MA, USA). Membranes were incubated with 1% bovine serum albumin (BSA) and then incubated with the indicated antibodies overnight at 4°C followed by incubation with an alkaline phosphatase-conjugated secondary antibody for 1 h. Proteins were visualized by incubating with the colorimetric substrates, NBT and BCIP.

### DNA fragmentation analysis

The phenol/chloroform/isoamyl alcohol procedure was used to extract DNA from aliquots of cell lysates (5 × 10^6^ cells per sample) that had been digested with proteinase. The DNA was ethanol-precipitated, dissolved in Tris-EDTA buffer, incubated with RNase A (50 μg/ml) for 30 min at 37°C, and then analyzed by electrophoresis in 1.5% agarose gels.

### Flow cytometric analysis of the cell cycle

Trypsinized cells were washed twice with ice-cold phosphate-buffered saline (PBS) and fixed in 70% ethanol at -20°C for overnight. After fixation, cells were washed twice with PBS and incubated in 1 ml of 0.5% Triton X-100/PBS containing 1 mg/ml of RNase A at 37°C for 30 min, followed by staining with 1 ml of 50 μg/ml propidium iodide (PI) for 10 min. Fluorescence emitted from the PI-DNA complex was quantitated after excitation of the fluorescent dye by FACScan flow cytometry (Becton Dickenson, San Jose, CA, USA). Ratios of cells at the G_2_/M and sub-G_1_ phases were measured and are expressed as percentages (%) of total counts.

### Measurement of the MMP

After different treatments, cells were incubated with 40 nM DiOC6(3) for 15 min at 37°C, then washed with ice-cold PBS, and collected by centrifugation at 500 x*g* for 10 min. Collected cells were resuspended in 500 ml of PBS containing 40 nM DiOC6(3). Fluorescence intensities of DiOC6(3) were analyzed on a flow cytometer (FACScan, Becton Dickinson) with excitation and emission settings of 484 and 500 nm, respectively.

### Detection of hypodiploid cells by EVO in RCC

Cells were plated in duplicate in 24-well plates, and then incubated for 24 h. The medium were changed, and different treatments were added to each well. Cells were treated for 12 h, and the supernatant and cells were harvested by exposing the cells to a 0.25%, Trypsin-EDTA solution for 10 min, then centrifugation, washing in phosphate-buffered saline (PBS), and fixation in 3 mL of ice-cold 100% ethanol. All samples were incubated for 30 min at room temperature in the dark. The cell cycle distribution and hypodiploid cells were determined using a FACScan Flow Cytometer (FACScan, Becton Dickinson).

### Tumor xenograft implantation

The studies described in this report were approved by the Animal Review Committee of Taipei Medical University Animal Studies. Athymic nude mice (nu/nu; 3-week-old males) were obtained from BioLASCO (Taipei, Taiwan) and acclimatized to laboratory conditions for 1 week before tumor implantation. Animals (5 mice/treatment group) were inoculated with a subcutaneous (s.c.) injection on the flank with human A498 RCC cells (10^7^ cells/mouse) in 0.2 ml of saline. Drug therapy was begun when tumors reached an average volume 80~100 mm^3^ (after 28~30 days). Treatments consisted of three intraperitoneal (i.p.) injections a week of EVO (30 mg/kg in 0.2 ml DMSO) over 2 weeks. Control animals received injections of DMSO. Tumors were measured three times per week, and volumes were calculated using the following formula: 1/2 x Length x Width^2^ [33]. Animals were killed by an i.p. injection of pentobarbital on day 46.

### Immunohistochemistry

Sections were deparaffinized in xylene, followed by ethanol, then blocking in 0.3% H_2_O_2_ for 30 min, and washing in Tris-buffered saline (TBS) three times. The heat-induced epitope retrieval water bath was set to 60°C, and slides were incubated in retrieval solution. The primary antibody which recognizes p-PERK was diluted in TBS with 1% BSA overnight at 4 °C. After washing with TBS three times, a section was incubated with a secondary antibody for 1 h, then developed with DAB, dehydrated, cleared, and covered with a coverslip and mounting medium.

### Statistical analysis

Values are expressed as the mean ± standard deviation (S.D.) of three independent experiments. The significance of the difference from the respective controls in each experiment was assayed using a one-way analysis of variance (ANOVA) with a post-hoc Bonferroni analysis when applicable, and *p* values of < 0.05 or < 0.01 were considered statistically significant.

## Results

### EVO inhibited the viability of human A498 RCC cells via apoptosis

**Fig 1:**
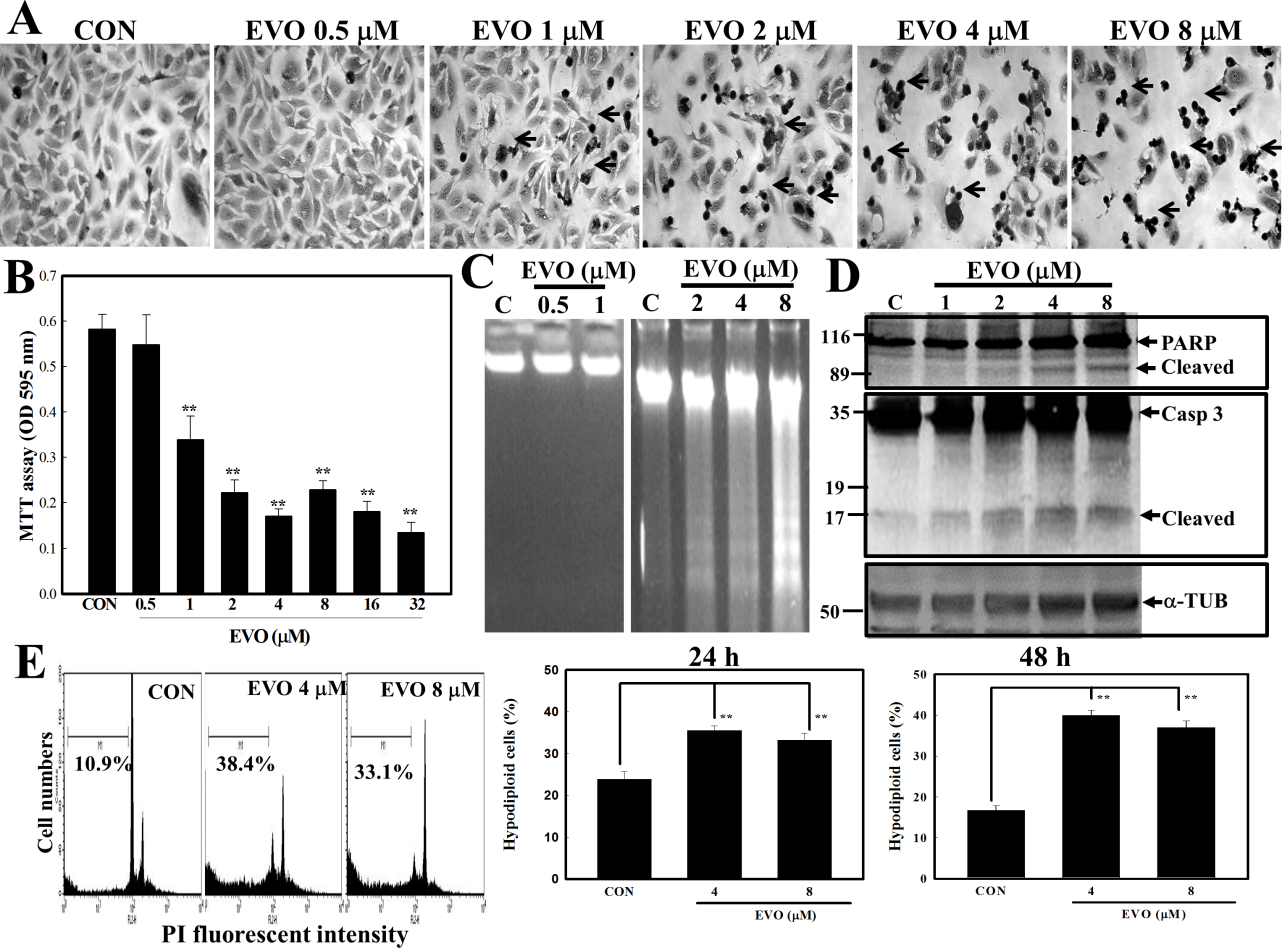
Evodiamine (EVO) reduction of viability of human A498 renal cell carcinoma (RCC) cells via apoptosis induction. (A) Alterations in cellular morphology by EVO (0.5, 1, 2, 4, and 8 μM) were observed microscopically via Giemsa staining. A498 cells were treated with different concentrations of EVO (0.5, 1, 2, 4, and 8 μM) for 12 h, and morphology of cells was observed microscopically. (B) EVO reduction of cell viability of A498 cells according to an MTT assay. A498 cells were treated with different concentrations of EVO (0.5, 1, 2, 4, 8, 16, and 32 μM) for 12 h, and viability of cells was examined by an MTT assay. (C) Loss of DNA integrity with increased DNA ladders by EVO (0.5, 1, 2, 4, and 8 μM) was examined by agarose electrophoresis. (D) EVO induction of cleavage of caspase (Casp)-3 and the PARP protein in A498 cells by Western blotting using specific antibodies. (E) Increased percentage of hypodiploid cells by EVO in A498 RCC cells. Cells were treated with EVO (4 and 8 μM) for 12 h, and the percentage of hypodiploid cells was examined by a flow cytometric analysis via propidium iodide (PI) staining. Each data point was calculated from triplicate determinations, and data are displayed as the mean ± S.D. ** *p*<0.01, significantly differs compared to the control (CON) group.

The chemical structure of EVO is a natural chemical isolated from *E*. *rutaecarpa*. First, we examined the morphological changes of human A498 cells by different concentrations of EVO. As shown in Fig 1A, morphological observations showed that EVO significantly altered the morphology of A498 cells, and dark rounded cells (Arrow) indicating chromatin-condensed cells were observed via Giemsa staining (Fig 1A). Examination of viability of A498 cells by MTT assay indicated that EVO reduced the viability of A498 cells in a concentration-dependent manner (Fig 1B). Apoptotic characteristics, including DNA ladders (Fig 1C) and cleavage of the Casp-3/PARP proteins (Fig 1D), were observed in EVO-treated A498 cells. Additionally, data of the flow cytometric analysis indicated that increased percentage of hypodiploid cells (in the sub-G_1_ phase) were detected in A498 cells after EVO treatment (Fig 1E). These results support EVO's inhibition of A498 RCC cell viability being mediated by apoptosis induction.

### Activation of JNK plays a critical role in EVO-induced apoptosis of A498 RCC cells

**Fig 2:**
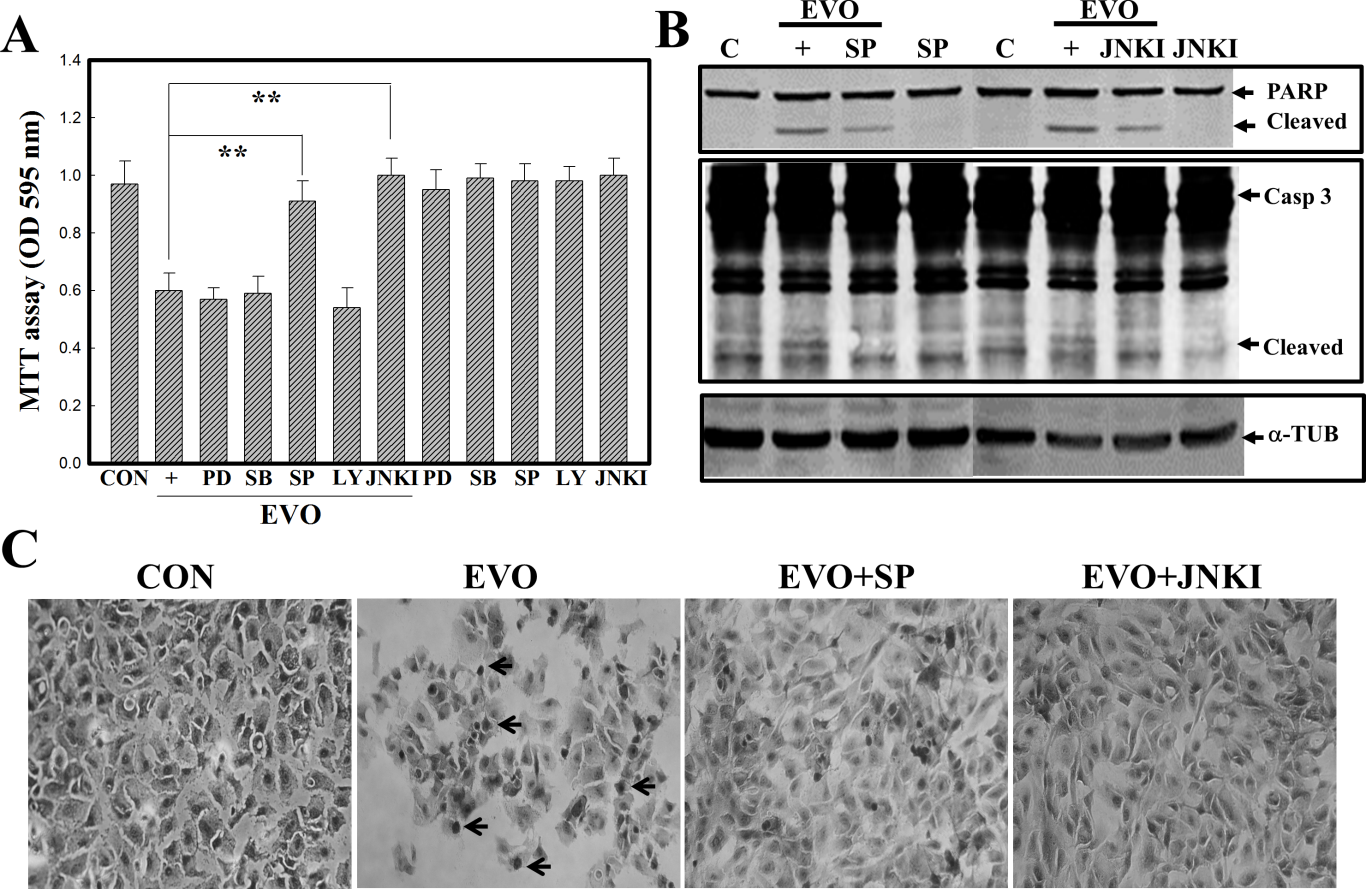
The c-Jun N-terminal kinase (JNK) inhibitors, SP600125 (SP) and JNKI, protect A498 cells from evodiamine (EVO)-induced apoptosis. (A) The JNK inhibitors, SP and JNKI, prevented EVO-induced cell death in human A498 renal cell carcinoma (RCC) cells. Cells were treated with the indicated kinase inhibitors (20 mM) for 30 min followed by EVO (4 mM) treatment for 12 h, and viability of cells under different treatments was evaluated by an MTT assay. (B) The JNK inhibitors, SP and JNKI, inhibited EVO-induced cleavages in caspase (Casp)-3 and poly(ADP ribose) polymerase (PARP) protein by Western blotting. (C) EVO-induced alternations in A498 morphology were reversed by the addition of the JNK inhibitors, SP and JNKI. As described above, the morphology of A498 cells was observed microscopically via Giemsa staining. Each data point was calculated from triplicate determinations, and data are displayed as the mean ± S.D. ** *p*<0.01, significantly differs between the indicated groups. Arrows indicate the chromatin-condensed cells.

MAPK members, including ERK, JNK, and p38, were reported to play roles in apoptosis. In order to elucidate the roles that MAPK members play in EVO-induced apoptosis of A498 RCC cells, pharmacological studies were performed using specific inhibitors of MAPK members, including the ERK inhibitor, PD98058 (PD), the p38 inhibitor, SB203580 (SB), the JNK inhibitors, SP600125 (SP) and JNKI, and the PI3K inhibitor (LY294002). As illustrated in Fig 2A, data of the MTT assay showed that the JNK inhibitors, SP and JNKI, significantly reduced EVO-induced cell death in A498 RCC cells. However, no inhibitors except JNK inhibitors showed any effect on EVO-induced cell death. Furthermore, the JNK inhibitors, SP and JNKI, additionally inhibited EVO-induced cleavage of the PARP and Casp-3 proteins in A498 cells according to a Western blot analysis (Fig 2B). No change in the α-TUB protein in each lane was described as an internal control to verify similar protein loadings. Morphological observations showed that EVO-decreased cell numbers and EVO-increased chromatin-condensed cells were reversed by the addition of the JNK inhibitors, SP and JNKI (Fig 2C). These results support the critical role played by JNK in EVO-induced apoptosis of A498 RCC cells.

### Disruption of the MMP with increased Bcl-2 protein phosphorylation at Ser-70 by EVO in A498 RCC cells

**Fig 3:**
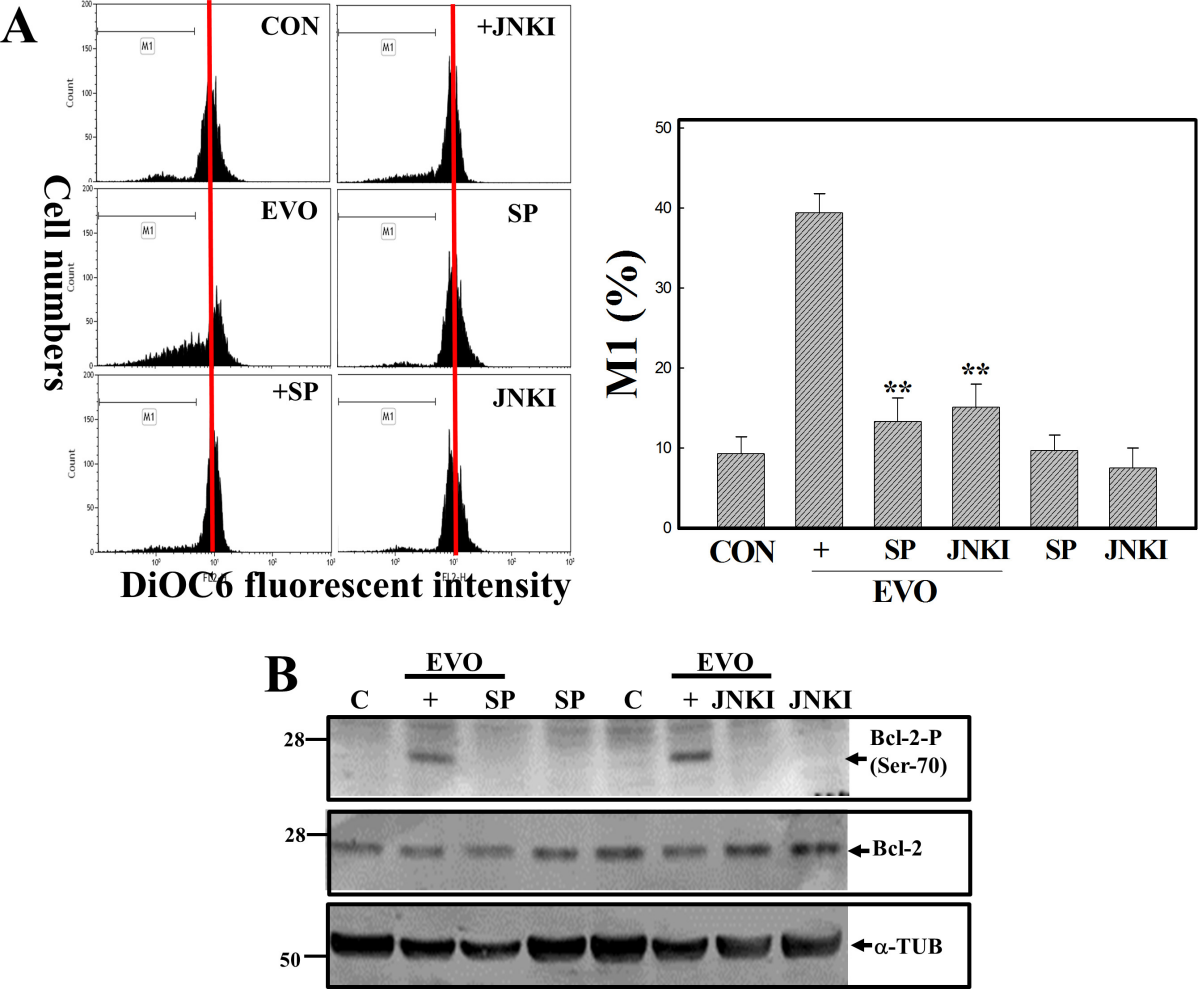
Disruption of the mitochondrial membrane potential (MMP) with an increase in the phosphorylation of the Bcl-2 protein in evodiamine (EVO)-treated human A498 renal cell carcinoma (RCC) cells, which was inhibited by adding the JNK inhibitors, SP600125 (SP) and JNKI. (A) Loss of the MMP by EVO was inhibited by addition of the JNK inhibitors, SP and JNKI, in A498 cells. Cells were treated with or without the JNK inhibitors, SP or JNKI, (20 μM) for 30 min followed by EVO (4 μM) treatment for an additional 12 h, and the MMP was detected by a flow cytometric analysis using DiOC6 as a fluorescent dye. (upper) A representative example of flow cytometric data is shown; (lower) quantification of the M1 ratio from three independent experiments is shown. (B) Altered expressions of Bcl-2 family proteins including Bcl-2, phosphorylated Bcl-2 (Ser-70), Bax, and the Mcl-1 protein by EVO were detected in A498 cells by Western blotting using specific antibodies. As described above, expression of the indicated protein was detected using specific antibodies. Each data point was calculated from triplicate determinations, and data are displayed as the mean ± S.D. ** *p*<0.01, significantly differs compared to the EVO-treated group.

Disruption of the MMP was shown to occur in the apoptotic process under various stimulations; however, the effect of EVO on the MMP in apoptosis of RCC cells is still unclear. The MMP of A498 RCC cells with or without EVO stimulation was examined by flow cytometry using DiOC6, a mitochondrial fluorescent dye. As depicted in Fig 3A, loss of the MMP was identified in A498 cells stimulated with EVO, and it was significantly inhibited by the addition of the JNK inhibitors, SP and JNKI. Bcl-2 family proteins were shown to regulate the MMP during apoptosis, and data in Fig 3B show that EVO altered expressions of antiapoptotic Bcl-2 family proteins including the Bcl-2 and Mcl-1 proteins, and the proapoptotic Bcl-2 family protein, Bax, in A498 RCC cells. Significantly, EVO treatment induced a higher level of the phosphorylated Bcl-2 protein at Ser-70 in A498 cells, which was inhibited by the addition of the JNK inhibitors, SP and JNKI.

### Structural importance of EVO leading to apoptosis of human A498 RCC cells

**Fig 4:**
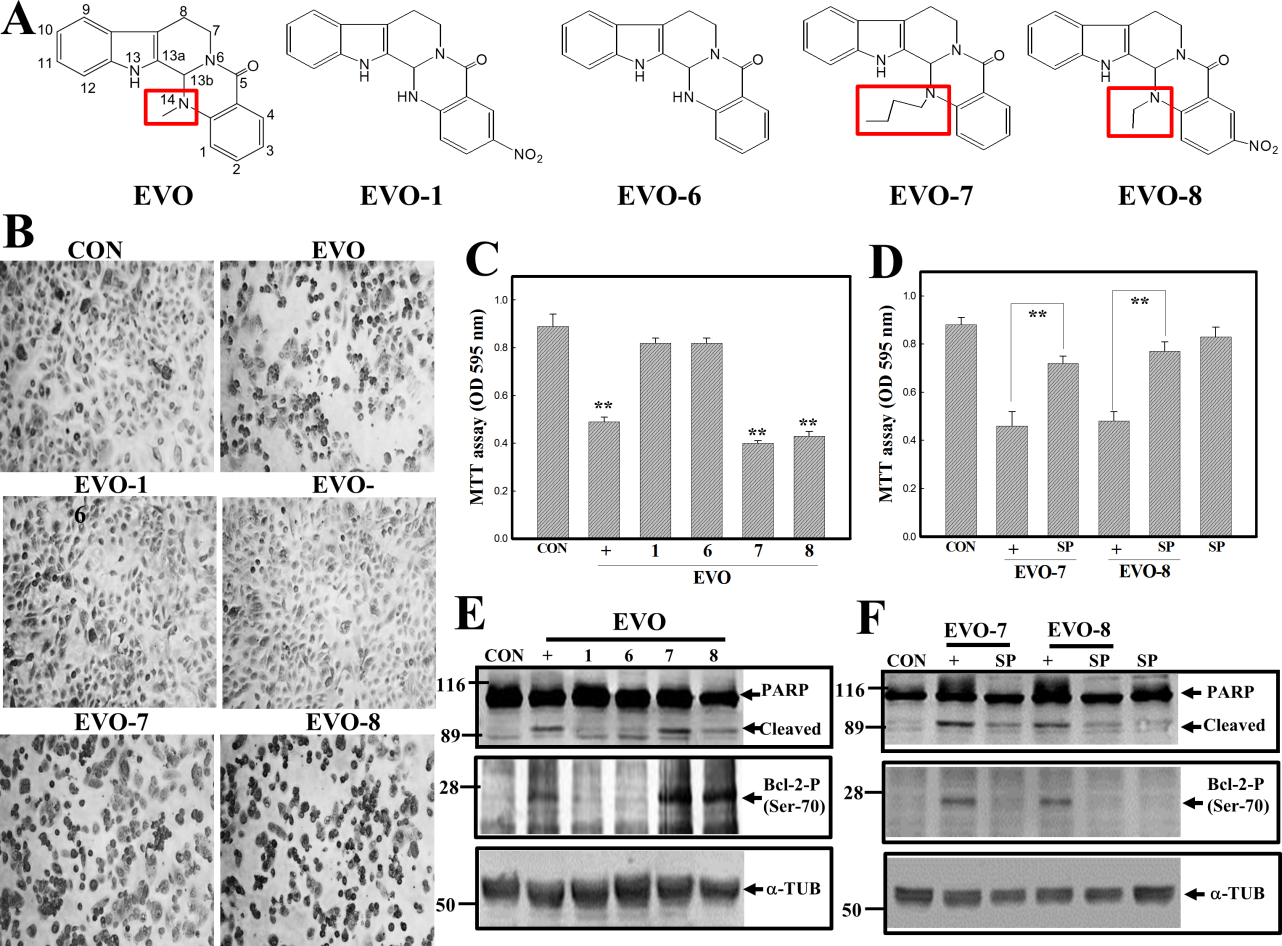
Structure-activity relationship of evodiamine (EVO) and related chemicals on apoptotic events in human A498 renal cell carcinoma (RCC) cells. (A) The chemical structures of EVO and structurally related chemicals including EVO-1, -6, -7, and -8 are depicted. (B) Alternative morphological changes in EVOs were observed microscopically. A498 cells were treated with the indicated EVOs (4 μM) for 12 h, and the morphology of cells was observed microscopically via Giemsa staining. (C) Differential cytotoxicity elicited by the EVOs in A498 cells. A498 cells were treated with the indicated EVOs (4 μM) for 12 h, and cytotoxicity induced by the EVOs was analyzed by an MTT assay. (D) The JNK inhibitor, SP600126 (SP), inhibited EVO-7- and EVO-8-induced cytotoxicity in A498 cells. Cells were treated with SP (20 μM) for 30 min followed by EVO stimulation for 12 h. Viability of cells under various treatments was examined by an MTT assay. (E) EVO, -7, and -8, but not EVO-1 or -6, induced poly(ADP ribose) polymerase (PARP) cleavage and phosphorylation of the Bcl-2 protein in A498 cells. (F) SP addition reduced PARP cleavage and phosphorylation of the Bcl-2 protein by EVO-7 and -8 in A498 cells. Each data point was calculated from triplicate determinations, and data are displayed as the mean ± S.D. ** *p*<0.01, significantly differs compared to the control group (C) or between indicated groups (D).

We showed that EVO at lower concentrations significantly reduced the viability of human A498 RCC cells, and it was interesting to elucidate the critical functional groups involved. In Fig 4A, EVO and four synthetic structure-related chemicals, including EVO-1, -6, -7, and -8, were used in the study. Analysis of the chemical structures of these EVOs indicated that EVO, EVO-7, and -8 possessed alkyl substitutions at C14, while EVO-1 and -6 did not. Detection of morphological alternations in A498 RCC cells under the indicated EVO treatment showed that EVO, -7, and -8 reduced cell numbers and increased chromatin-condensed cells under microscopic observations via Giemsa staining (Fig 4B). Data of the MTT assay showed significant decreases in the viability of A498 cells in the presence of EVO, -7, and -8 treatment, however not in the presence of EVO-1 or -6 (Fig 4C). The addition of the JNK inhibitor, SP, significantly protected A498 cells from EVO-and EVO-8 induced cell death according to the MTT assay (Fig 4D). Data of Western blotting showed that increased cleavage of the PARP protein and expression of the phosphorylated Bcl-2 protein were detected in EVO-, EVO-7-, and EVO-8-treated A498 cells, and those were suppressed by adding the JNK inhibitor, SP (Fig 4E and 4F).

### Activation of PERK contributes to EVO-induced apoptosis of human A498 RCC cells

**Fig 5:**
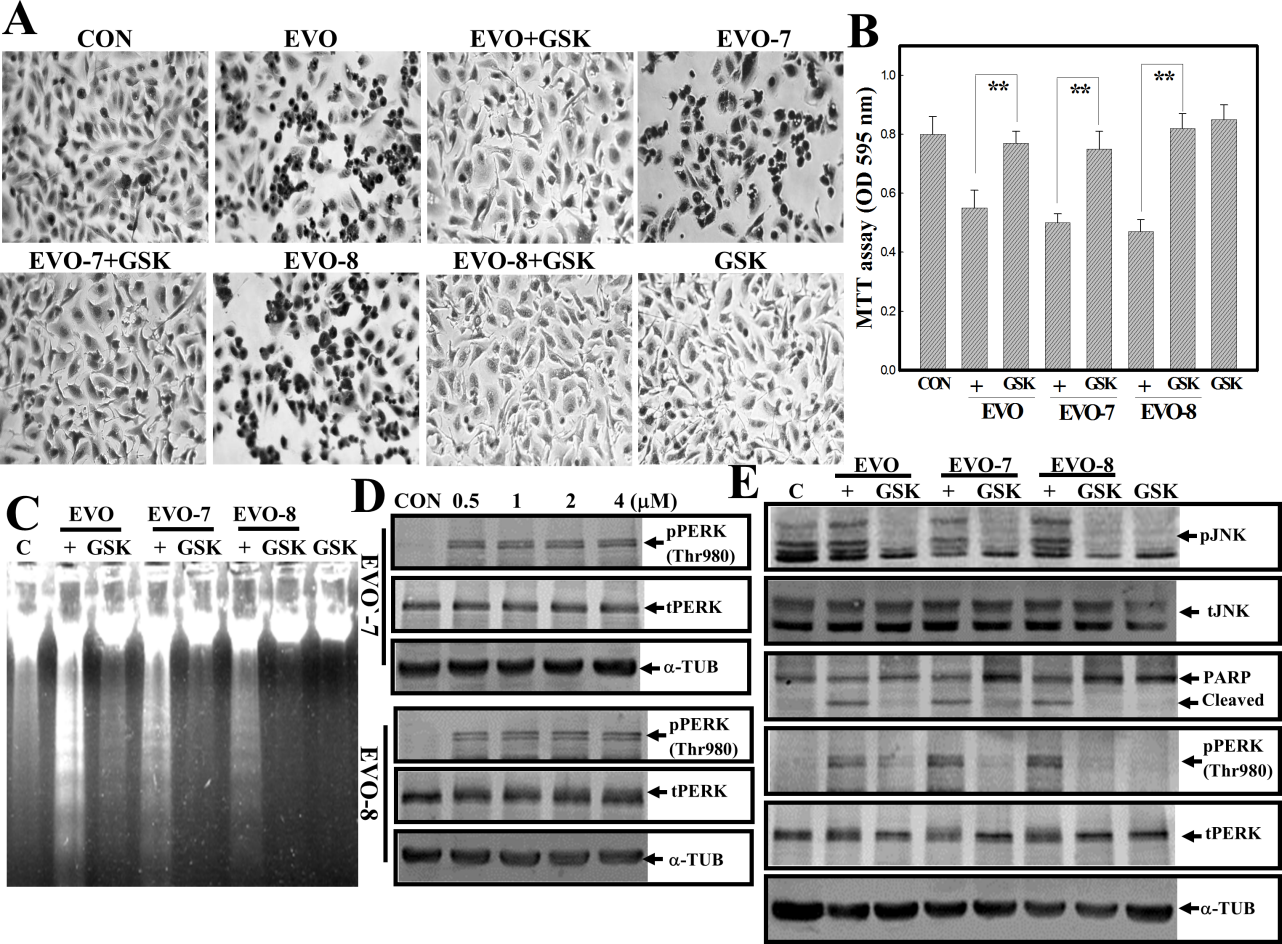
Activation of protein kinase RNA-like endoplasmic reticular kinase (PERK) contributes to evodiamine (EVO)-induced apoptosis of human A498 renal cell carcinoma (RCC) cells. (A) The PERK inhibitor, GSK, reversed morphological changes by EVOs including EVO, -7, and -8 in A498 cells. A498 cells were treated with or without GSK (20 mM) for 30 min followed by the indicated EVOs (4 μM) for 12 h. The morphology of A498 cells under different treatments was observed microscopically via Giemsa staining. (B) GSK protects A498 cells from EVO-induced cytotoxicity according to an MTT assay. As described in (A), the viability of cells was detected by an MTT assay. (C) GSK addition inhibits EVO-induced DNA ladder formation in A498 cells. As described in (A), the DNA integrity was examined by agarose electrophoresis. (D) EVO-7 and -8 concentration-dependently increased phosphorylation of PERK at Thr980 (p-PERK Thr980) in A498 cells. Cells were treated with different concentrations of EVO-7 or -8 for 12 h, and expressions of phosphorylated and total PERK were examined by Western blotting using specific antibodies. (E) GSK inhibited EVO-, EVO-7-, and EVO-8-induced phosphorylation of c-Jun N-terminal kinase (JNK), PERK, and Bcl-2 proteins in A498 cells. As described before, expressions of indicated proteins were examined by Western blotting using specific antibodies. Each data point was calculated from triplicate determinations, and data are displayed as the mean ± S.D. ** *p*<0.01, significantly differs between the indicated groups (B).

PERK, an ER stress protein, was shown to regulate the viability of cells, and therefore we investigated if PERK activation is involved in EVO-induced apoptosis of human A498 RCC cells. GSK2606414 (GSK), a specific PERK inhibitor, was used in this study to examine the role of PERK in EVO-induced apoptosis. Morphological observations indicated that EVO-, EVO-7- and EVO-8-induced chromatin-condensed cells were inhibited by the addition of GSK according to Giemsa staining (Fig 5A). Data of the MTT assay showed that decreases in the viability of A498 cells by EVO, -7, and -8 were significantly reversed by the addition of GSK (Fig 5B). Examination of DNA integrity via agarose electrophoresis indicated that DNA ladders induced by EVO, -7, and -8 were suppressed by the addition of GSK (Fig 5C). When A498 cells were treated with different concentrations of EVO-7 and EVO-8, increased expression of pPERK at Thr-980 was detected in a concentration-dependent manner (Fig 5D). Increased expression of pJNK, pPERK, and cleavages of PARP protein by EVO, EVO-7, and EVO-8 was suppressed by addition of the PERK inhibitor GSK in human A498 RCC cells (Fig 5E).

### Antitumor activity of EVO against human RCC cells in vivo

**Fig 6:**
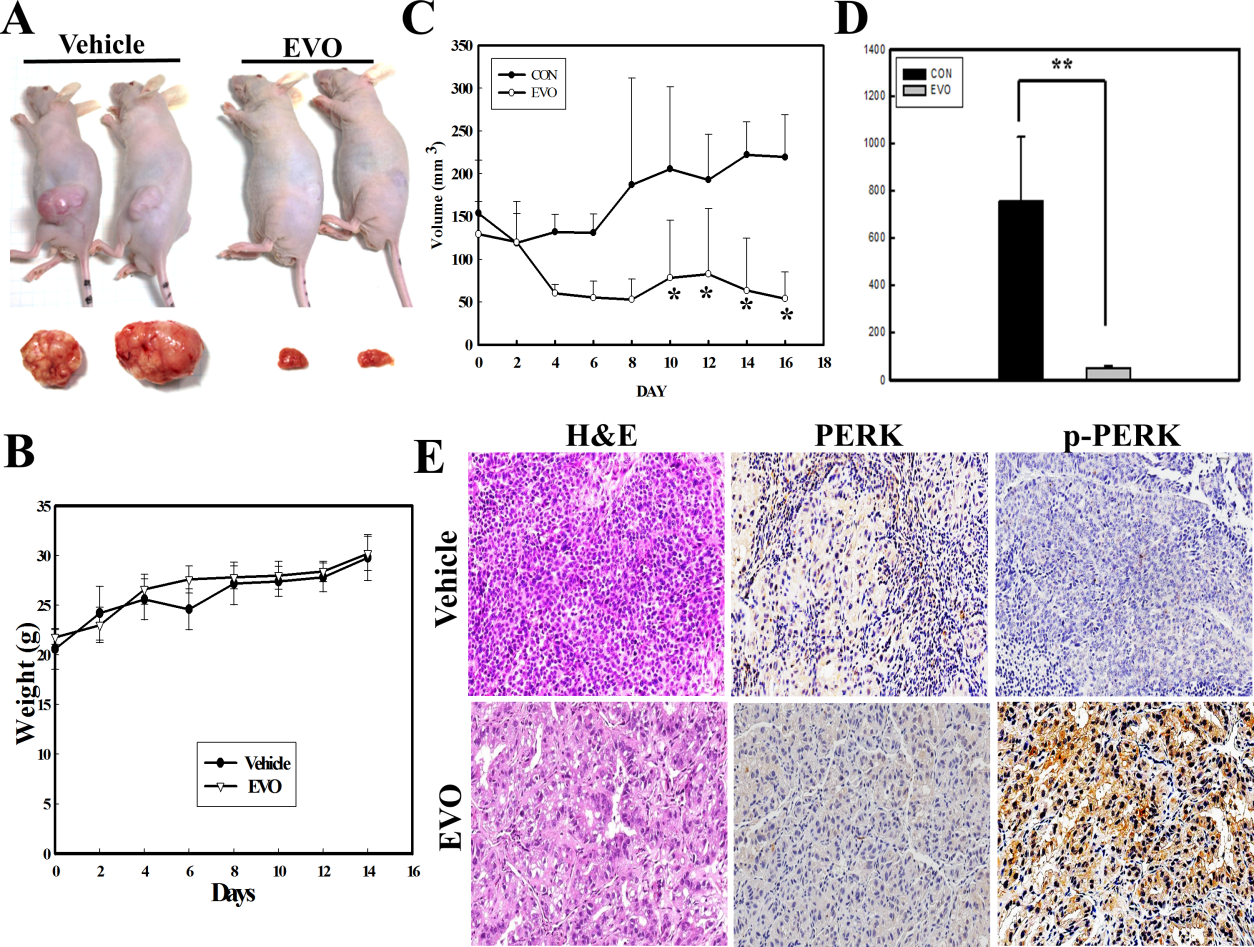
In vivo antitumor activity of evodiamine (EVO) against renal cell carcinoma (RCC) growth elicited by a subcutaneous injection of A498 cells with increased phosphorylated protein kinase RNA-like endoplasmic reticular kinase (PERK) protein. (A) A representative example of tumors derived from the control (phosphate-buffered saline (PBS); vehicle) and EVO-treated groups at the end the experiments are shown. (B) No difference in the body weight of mice between the control (vehicle) and EVO-treated groups was observed. (C) EVO treatment significantly inhibited in vivo RCC growth in nude mice. The in vivo study is described in "Materials and Methods", and tumor volumes were measured. (D) EVO significantly reduced tumor weights elicited by A498 cells in vivo at the end of the experiment. At the end of the experiment (18 days), tumors were excised and weighed from the control and EVO-treated groups. (E) Tumor specimens were stained with H&E to identify tumor locations, and expressions of total (t-PERK) and phosphorylated PERK (p-PERK) protein in tumor sections of the control and EVO-treated groups were detected by immunohistochemistry using specific antibodies. Each data point was calculated from triplicate determinations, and data are displayed as the mean ± S.D (n = 5). ** *p*<0.01, significantly differs between the indicated groups (D).

All animal works have been conducted according to relevant national and international guidelines. In order to evaluate the in vivo anti-RCC effect of EVO, an s.c. injection of A498 cells in nude mice followed by an i.p, injection of EVO (30 mg/kg) or PBS (vehicle) was given three times a week for 3 weeks. A representative example of tumors excised from nude mice after EVO or vehicle injection is shown in Fig 6A, and tumor weights were measured (*n* = 5) at the end of the experiments. Comparison of body weights of nude mice showed no difference between the EVO- and vehicle-treated groups (Fig 6B). Measuring the tumor volume elicited by A498 RCC cells showed time-dependent increases in the tumor volume in the vehicle-treated group, and EVO injections significantly reduced the volume (Fig 6C). Measuring the tumor weight at the end of the experiments showed that EVO treatment significantly reduced the tumor weight in vivo (*n* = 5) (Fig 6D). Immunohistochemical data showed that increased expression of the p-PERK protein was detected in tumor tissues under EVO treatment (Fig 6E). In vivo anti-RCC growth by EVO with increased p-PERK protein expression was demonstrated herein.

### EVO induces apoptosis in various human renal cell carcinoma cells including 786-O, ACHN, and Caki-1

**Fig 7:**
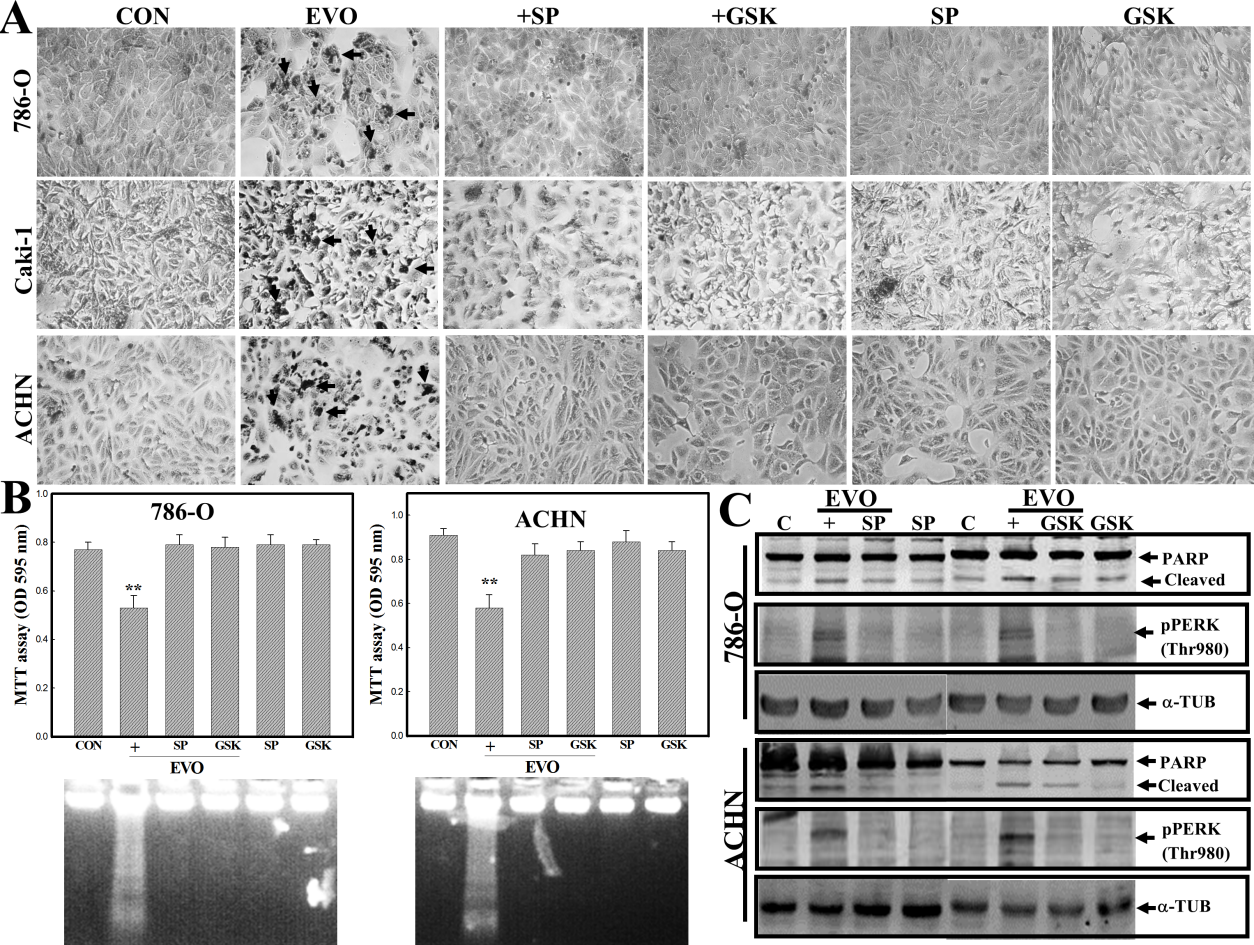
Effects of EVO on various human renal carcinoma cells including 786-O, ACHN, and Caki-1. (A) The apoptotic morphology of EVO-treated 786-O, ACHN, and Caki-1 cells. These cells were treated with or without EVO (4 μM) in the presence or absence of SP or GSK (20 μM) for 12 h, and the morphology of cells was observed by Giemsa staining under microscopy. (B) SP and GSK inhibited EVO-induced cell death and DNA ladders in 786-O and ACHN cells. Human RCC cells 786-O and ACHN were treated as described in (A), and viability of cells and DNA integrity were analyzed by MTT assay (upper panel) and agarose electrophoresis (lower panel), respectively. (C) SP and GSK inhibited EVO-induced cleavages of PARP protein and phosphorylated PERP protein in 786-O and ACHN cells. As described in (A), expression of PARP, pPERK, and α-TUB protein was examined by Western blotting using specific antibodies. Each data point was calculated from three triplicate groups, and data are displayed as the mean ± S.D. **p<0.01, significantly differs from the control (CON) groups.

In order to identify if EVO induces apoptosis in other types of human RCC cells, three additional RCC cells, including 786-O, ACHN, and Caki-1, were used. In S1 Fig, EVO reduced the viability of human RCC cells 786-O, ACHN, and Caki-1 in a concentration-dependent manner. As shown in Fig 7A, increased chromatin-condensed cells occurred in EVO-treated 786-O, ACHN, and Caki-1 cells according to Giemsa staining under microscopic observation, and those were inhibited by addition of JNK inhibitor SP or PERK inhibitor GSK. Data of the MTT and DNA integrity assay showed that EVO-induced DNA ladders and–reduced viability of 786-O and ACHN cells were reversed by addition of SP and GSK, respectively (Fig 7B). Results of Western blotting indicated that EVO-induced cleavages of PARP and phosphorylated PERP protein were inhibited by SP and GSK in 786-O and ACHN cells (Fig 7C). Extensive apoptotic effects elicited by EVO against the viability of various human RCC cells were demonstrated.

## Discussion

**Fig 8:**
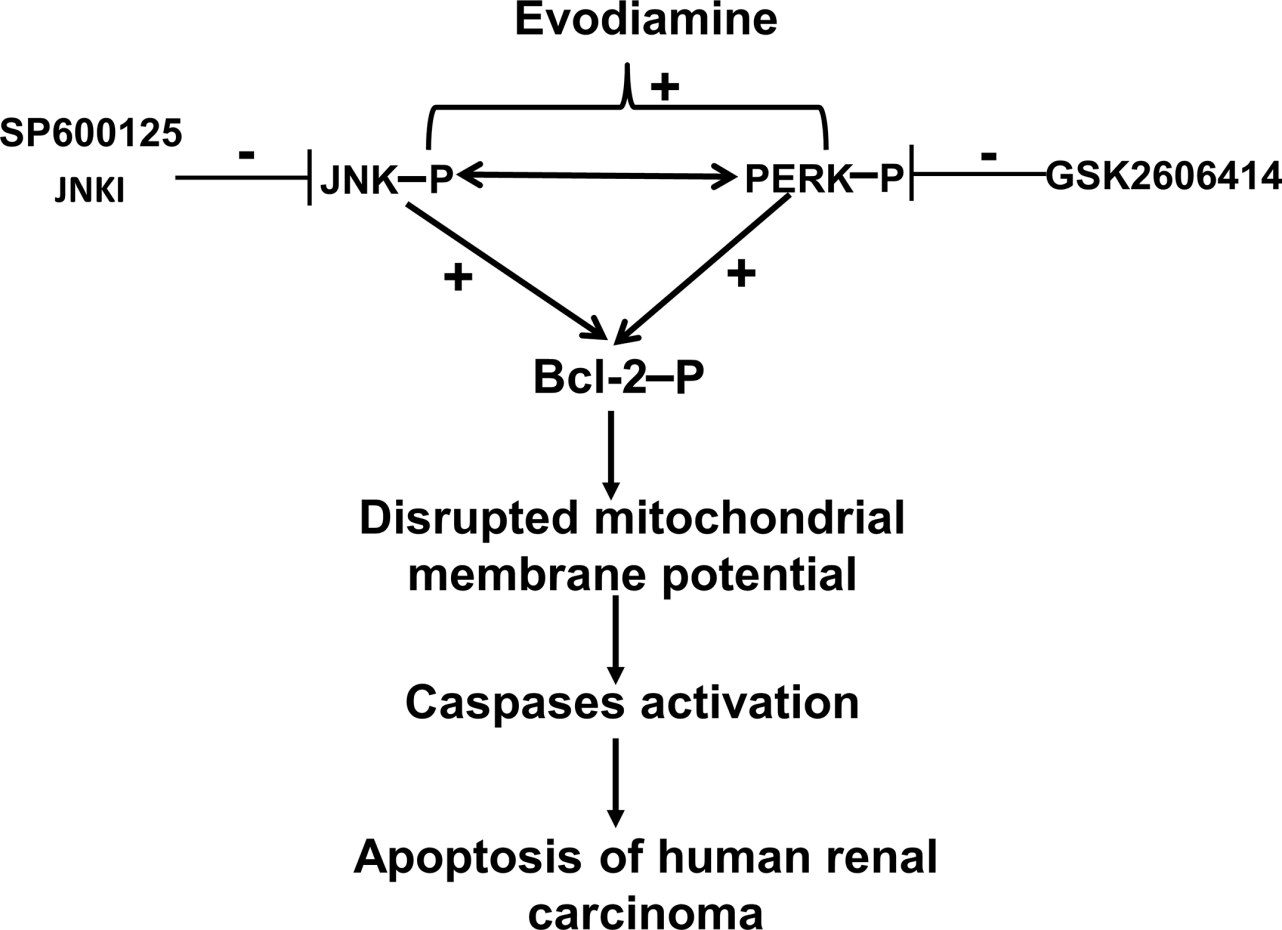
A tentative mechanism of evodiamine (EVO)-induced apoptosis in human A498 renal cell carcinoma (RCC) cells is depicted. It indicates that EVO induces phosphorylation of c-Jun N-terminal kinase (JNK) and protein kinase RNA-like endoplasmic reticular kinase (PERK) leading to disruption of the mitochondrial membrane potential which in turn activates caspases to cause the apoptosis of human RCC cells.

In this study, the antitumor mechanism of EVO against human A498 RCC cells was investigated. To elucidate if JNK or PERK participate in EVO-induced apoptosis and mitochondrial dysfunction, the influence of JNK and PERK inhibitors on EVO-induced events was studied for the first time. Results showed that EVO significantly reduced the viability of human A498 RCC cells with increased JNK and PERK protein phosphorylation. Pharmacological suppression of JNK and PERK phosphorylation reversed EVO-induced apoptosis and loss of the MMP associated with decreased expression of the phosphorylated Bcl-2 protein (Ser-70) of A498 cells. In vivo study showed that EVO suppression of RCC growth in mice with elevated phosphorylation of the PERK protein. Apoptosis by EVO was also demonstrated in other RCC cells such as 786-O, ACHN, and Caki-1 cells. The antitumor mechanism of EVO against RCC cells with increased JNK/PERK-mediated phosphorylation of the Bcl-2 protein leading to disruption of the MMP was demonstrated herein (Fig 8).

JNK was reported to function as a proapoptotic kinase in various cells under chemical stimulation. Apoptosis induced by Nerve growth factor (NGF) withdrawal was suppressed by blocking the JNK cascade in PC-12 cells [34, 35], and genetic knockdown experiments showed that JNK has a proapoptotic role in various cells. The key evidence to suggest JNK's activation in apoptosis is the observation that JNK-/- mice showed resistance to apoptosis induced by irradiation and chemicals [36, 37]. Although activation of the JNK pathway may contribute to apoptosis by certain stimuli, the underlying mechanism is still unclear. Our previous study indicated that activation of JNK participated in EVO-induced apoptosis and G_2_/M arrest in human colorectal cancer cells. In the present study, EVO induced apoptosis of human A498 RCC cells with increased JNK protein phosphorylation, and the addition of the JNK inhibitors, SP600125 and JNKI, significantly prevented EVO-induced apoptosis. These results support the proapoptotic actions of JNK activation against human RCC cells by EVO.

ER stress is one of the pathways by which cell apoptotic signals are conducted in response to stimuli via accumulation of misfolded proteins leading to ER dysfunction. Three branches of ER stress, PERK, IRE1, and ATF6, showed their uniqueness in conducting survival or death signals, and that PERK is a central regulator in deciding the proapoptotic or antiapoptotic fates by interacting with different downstream molecules. Activation of PERK might phosphorylate downstream substrates such as translation initiation factor 2α (eIF2α), transcriptional factors FOXOs, and nuclear factor erythroid-derived 2 transcription factor (Nrf2) to regulate cell survival/apoptosis. In myocytes, deletion of PERK exhibited protective effects against apoptosis elicited by high glucose levels. Zhang et al.[38] indicated that activation of the PERK pathway is involved in nitric oxide (NO)-induced apoptosis of endothelial cells. Increased sensitivity of mutant ERBB2-expressing cells to ER stress relied on the PERK pathway to upregulate the proapoptotic cell surface receptor, TRAIL-R2, and activate the proapoptotic Casp-8. In the present study, EVO-induced apoptosis of A498 RCC cells was accompanied by increased PERK protein phosphorylation. GSK2606414 (GSK) was the first reported small-molecule inhibitor with high specificity for PERK. We found that application of GSK significantly reduced apoptosis with decreased PERK protein phosphorylation by EVO in A498 cells. We demonstrated that the proapoptotic effects of PERK contributed to EVO-induced apoptosis in human A498 RCC cells.

Evidence indicates that antiapoptotic functions of BcI-2 can be regulated by its phosphorylation. Hyperphosphorylation of the Bcl-2 protein by paclitaxel and other microtubule-disruptors is dependent on targeting microtubules which in turn cause mitotic arrest and apoptosis. JNK is one of the kinases implicated in induction of Bcl-2 protein phosphorylation. Lorin et al. [39] reported that JNK activation promotes Bcl-2 phosphorylation in 2-ME-mediated autophagy and apoptosis. Our previous study demonstrated that EVO induced tubulin polymerization and G_2_/M arrest in human CRC cells. In human A498 RCC cells, EVO stimulation induced JNK protein phosphorylation and Bcl-2 protein phosphorylation at Ser-70, and suppression of JNK protein phosphorylation by JNK inhibitors significantly protected A498 cells from EVO-induced apoptosis, along with decreased Bcl-2 protein phosphorylation. A contribution of JNK-mediated Bcl-2 protein phosphorylation to EVO-induced apoptosis was indicated herein. The role of PERK activation in JNK and Bcl-2 protein phosphorylation leading to apoptosis is still undefined. In this study, we found that GSK suppression of PERK protein phosphorylation inhibited JNK and Bcl-2 protein phosphorylation stimulated by EVO in A498 cells. This indicates that PERK may directly or indirectly stimulate JNK and Bcl-2 protein phosphorylation leading to apoptosis by EVO in human A498 RCC cells.

Our previous studies demonstrated that EVO-related chemicals containing an alkyl group such as methyl, ethyl, or butyl at position 14 exhibited to a significant apoptotic effects as EVO against the viability of human colon and ovarian cancer cells [13, 40]. In order to verify the contribution of structural substitutions of EVO in RCC cell apoptosis, PERK activation, and Bcl-2 protein phosphorylation, four EVO-related chemicals, including EVO-1, -6, -7, and -8, were applied in this study, and all chemicals shared the same chemical structure except for different substitutions including a methyl of EVO, an ethyl of EVO-8, a hydrogen of EVO-1 and -6, and a butyl of EVO-7 at position 14. Our results showed that EVO, -7, and -8, but not EVO-1 or -6, significantly reduced the viability of A498 cells via apoptosis induction with increased Bcl-2 phosphorylation and phosphorylated PERK protein expressions. Apoptosis elicited by EVO-7 and -8 was inhibited by the JNK inhibitor, SP600125, and the PERK inhibitor, GSK. Similar results by Ogasawara et al. indicated the critical role of a methyl group at position 14 of EVO in inhibiting invasion were with of Lewis lung cancer and melanoma cells [41]. Contribution of alkyl substitutions, such as methyl and butyl at position 14, to apoptosis induction and increased phosphorylated PERK and Bcl-2 protein by EVO against human RCC cells were demonstrated.

Our previous study demonstrated that EVO induced tubulin polymerization leading to G2/M arrest in human colorectal carcinoma cells [42]. In the present study, EVO at the concentration of 1 μM induced decreases in the proliferation of A498 cells via MTT assay with increased G2/M ratio as taxol did in A498 cells (Data not shown). At this condition, no obvious DNA ladder and chromatin condensation was observed as shown in Fig 1. It suggests that EVO possessed ability to induce G2/M arrest and apoptosis in human RCC cells, and the dependence between apoptosis and G2/M arrest by EVO needs to be further identified. Taken together, this study shows that EVO induces apoptosis in human A498 RCC cells, and reduces tumor growth elicited by A498 cells in vivo. EVO induces phosphorylation of JNK, PERK, and Bcl-2 proteins associated with disruption of the mitochondrial membrane potential and induced apoptosis in A498 cells. Inhibition of JNK and PERK reversed EVO-induced apoptosis, phosphorylation of the Bcl-2 protein, and loss of the mitochondrial membrane potential in A498 cells. A structure-activity study showed that a methyl at position 14 is important for EVO's action against the viability of human A498 RCC cells. The in vivo study using nude mice showed that EVO significantly reduced tumor growth and increased phosphorylation of the PERK protein in EVO-treated tumors. A new insight into the role of EVO as a potential anticancer agent for treatment of human renal carcinoma via stimulated JNK- and PERK-mediated phosphorylation of the Bcl-2 protein leading to disruption of the mitochondrial membrane potential was provided herein.

## Supporting Information

S1 Fig EVO dose-dependently inhibits the viability of human RCC cells including ACHN, 786-O, and Caki-1. (PPT)

## Abbreviations

Bcl-2: B-cell lymphoma 2
Casp: caspase
ER: endoplasmic reticulum
EVO: evodiamine
GSK: GSK2606414
JNK: c-Jun N-terminal kinase
MAPK: mitogen-activated protein kinase
MMP: mitochondrial membrane permeability
MTT: 3-(4,5,-dimethylthiazol)-2-yl-2,5-diphenyltetrazolium bromide
PARP: poly(ADP ribose) polymerase
PERK: protein kinase RNA-like endoplasmic reticulum kinase
PI3K: phosphoinositide 3-kinase
RCC: renal cell carcinoma
SDS: sodium dodecylsulfate
SP: SP600125
