# Supplementary material for: Protein Kinase RNA-Like Endoplasmic Reticulum Kinase-Mediated Bcl-2 Protein Phosphorylation Contributes to Evodiamine-Induced Apoptosis of Human Renal Cell Carcinoma Cells
Source: PLoS One. 2016 Aug 2;11(8):e0160484. doi: 10.1371/journal.pone.0160484 (PMC4970736; doi:10.1371/journal.pone.0160484)
Supplement: S1 Fig — (PPT) [file pone.0160484.s001.ppt]

## Slide 1
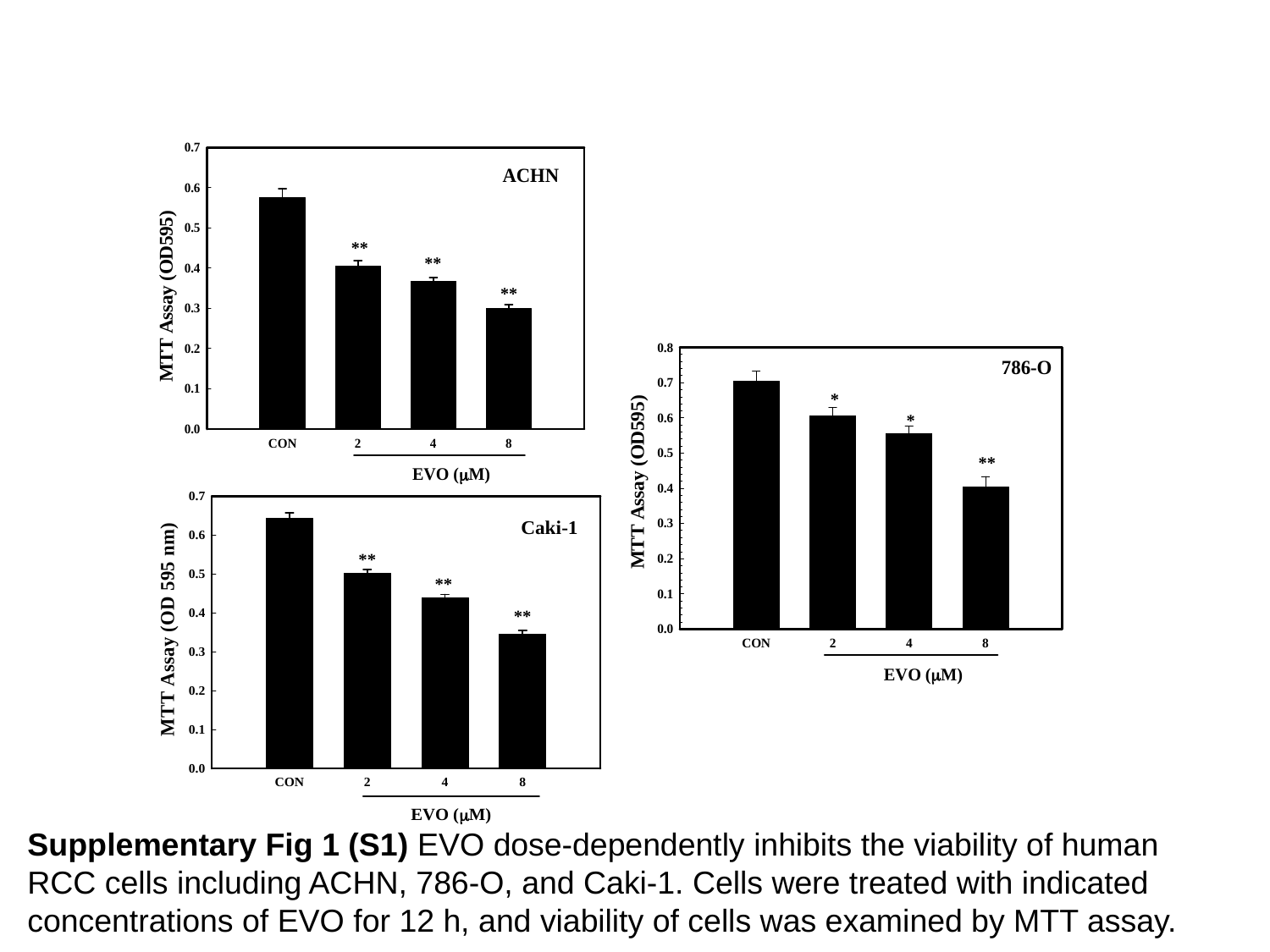

Supplementary Fig 1 (S1) EVO dose-dependently inhibits the viability of human RCC cells including ACHN, 786-O, and Caki-1. Cells were treated with indicated concentrations of EVO for 12 h, and viability of cells was examined by MTT assay.
